# Pelvic congestion syndrome and embolization of pelvic varicose veins

**DOI:** 10.1590/1677-5449.190061

**Published:** 2019-11-08

**Authors:** Mateus Picada Corrêa, Larissa Bianchini, Jaber Nashat Saleh, Rafael Stevan Noel, Julio Cesar Bajerski

**Affiliations:** 1 Instituto Vascular de Passo Fundo – INVASC, Passo Fundo, RS, Brasil.

**Keywords:** varicose veins, venous insufficiency, embolization

## Abstract

Pelvic congestion syndrome (PGS) is defined as chronic pelvic pain for more than 6 months associated with perineal and vulvar varicose veins caused by reflux or obstruction in gonadal, gluteal, or parauterine veins. PGS accounts for 16-31% of cases of chronic pelvic pain, and is usually diagnosed in the third and fourth decades of life. Interest in this condition among vascular surgeons has been increasing over recent years because of its association with venous insufficiency of the lower limbs. Despite its significant prevalence, PGS is still poorly diagnosed in both gynecology and angiology offices. Therefore, in this article we review the etiology and diagnosis of this condition and the outcomes of the different types of treatment available.

## INTRODUCTION

The existence of pelvic varicose veins was first described by Richet, in 1857, and the term pelvic venous congestion syndrome was coined by Taylor^1^ in 1949.

Pelvic congestion syndrome (PCS) is defined as chronic pelvic pain for more than 6 months combined with varicose veins of the perineum or vulva, resulting from reflux or obstruction of the gonadal, gluteal, or parauterine veins. Around 16-31% of cases of chronic pelvic pain are caused by PCS,^2^ which is predominantly diagnosed during the third and fourth decades of life.^3^ Interest in this pathology has increased among vascular surgeons over recent years because of its association with lower limb venous insufficiency.

Although prevalent, PCS is still underdiagnosed at gynecological consultations and by angiologists and vascular surgeons. In this article, we review etiology and diagnosis of this pathology and the results of the several different types of treatment available.^4^

## ANATOMY

**Figure 1:**
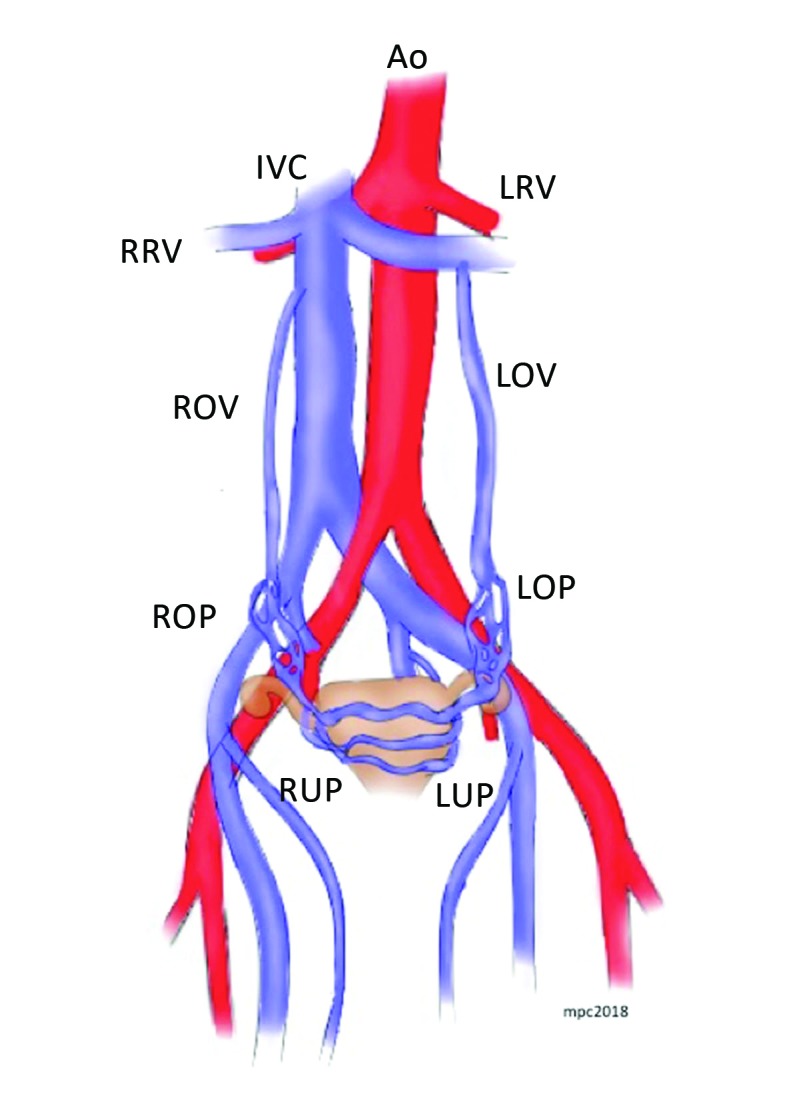
Schematic illustration of the anatomy of the pelvic veins. Ao = aorta; IVC = inferior vena cava; = LRV left renal vein; RRV = right renal vein; LOV= left ovarian vein; ROV= right ovarian vein; ROP = right ovarian plexus; LOP= left ovarian plexus; RUP = right uterine plexus; LUP = left uterine plexus.

The venous system of the uterus and ovaries drains to the internal iliac and gonadal veins. The pudendal veins receive the parietal tributaries and the visceral tributaries from the gonadal and vesicovaginal plexus and drain to the internal iliac veins. The ovarian veins drain the parametrium, the cervix, the mesosalpinx, and the pampiniform plexus, forming a rich venous-anastomotic plexus.^5^ The left ovarian vein is formed by union of two or three tributaries that meet at the level of the fourth lumbar vertebra, draining into the left renal vein on the left and the inferior vena cava on the right (Figure 1).^3^ In 10% of cases, the right ovarian vein drains to the right renal vein.^3^ The average diameter of the ovarian veins is less than 5 mm,^6^ and in 15% of cases the left gonadal vein does not have valves.^3^ However, when valves do exist, they are predominantly found in the distal portion of the vein. Heinz and Brenner^7^ conducted a study with 31 cadavers and found one case of pelvic varicose veins in a subject with valves, while venous dilation was not present in any of the 15 individuals without valves. However, gonadal vein valve incompetence may be present in up to 40% of cases.^8^

## PATHOPHYSIOLOGY

There are two etiological classifications of pelvic varicose veins. One of them classifies these varicose veins into three types: type 1, due to vein wall pathology, such as valve incompetence, valve agenesis, or malformations; type 2, secondary to vascular compression, such as in nutcracker syndrome (NCS), May-Thurner Syndrome (MTS), or collateralization secondary to post-thrombotic disease; and type 3, secondary to local extrinsic compression caused by pathologies such as endometriosis or tumor masses.^9^ The second classification describes four disorders: vulvar varicose veins without PCS, isolated hypogastric vein insufficiency, primary reflux of the gonadal veins, and pelvic collateralization secondary to compressive syndromes or extrinsic compression.^3^

In PCS with primary causes, varicose veins are the result of reflux caused by incompetent valves or degeneration of the vein wall. Reflux may also be the result of compression of the left renal vein by the superior mesenteric artery, compression of the left internal iliac vein in MTS, or malposition of the uterus,^10^ or may be a result of changes to the flow pattern caused by upstream venous hypertension.^8^ In men, varicose veins can cause varicocele.^11^ Daugherty and Gillespie^12^ found moderate to severe left common iliac vein compression in 18 patients and a high degree of stenosis of the suprarenal inferior vena cava in one patient. In all patients, pelvic symptoms were the predominant complaint and improved after stenting. There are also reports of some degree of iliac vein stenosis in up to 80% of patients with pelvic venous insufficiency.^13^

The etiology of primary reflux has not yet been fully explained. It is estimated that it involves a genetic component in up to 50% of patients.^14^ A hormonal factor also appears to contribute to the condition, to the extent that estradiol induces selective dilation of the ovarian and uterine veins during pregnancy, putting greater stress on the valves.^15^ Indeed, up to 50% of women with PCS have polycystic ovaries identifiable by echography, without hirsutism or amenorrhea.^16^ The pelvic pain is caused by blood stasis in the dilated pelvic veins, which can activate pain receptors in the walls of vessels, in addition to provoking release of neurotransmitters and substance P.^14^

## CLINICAL PRESENTATION

Classically, the most prevalent symptom is pelvic pain, which may be accompanied by dysmenorrhea, dyspareunia, and bladder irritation. Physical examination reveals vulvar varicosities, suprapubic varicose veins, and varicose veins on the posterior surfaces of the thighs.^3^ Mahmoud et al.^17^ conducted a review of 20 studies, finding that dysmenorrhea was reported in 86% (18.4-100%) of cases; while other frequent symptoms were dyspareunia (40.8%), lower limbs varicosities (58.7%), and vulvar varicosities (45.9%). Sensitivity to palpation of the ovaries and dyspareunia had 94% sensitivity and 77% specificity for PCS.^18^

After ruling out other more common causes of chronic pelvic pain including endometriosis, pelvic inflammatory disease, interstitial cystitis, and leiomyomas, pelvic ultrasound is employed to view the gonadal vessels.^19^

The association between PCS and lower limb venous insufficiency was demonstrated in a study conducted in Turkey, which showed that PCS was the cause of chronic pelvic pain in 30% of 100 consecutive patients, and that 70% of these cases also had symptoms of lower limb venous insufficiency, with reflux of the common femoral vein being the most frequent finding.^20^

## IMAGING EXAMS

The first imaging exam performed tends be transvaginal pelvic echography.^21^ The extent of pelvic vein dilation associated with pelvic pain is variable, although 4 mm is considered normal, 4-8 mm is associated with asymptomatic reflux, and measurements > 8 mm are associated with reflux and symptoms.^22^ Therefore, findings of dilated ovarian veins with diameter exceeding 8 mm, or parauterine veins > 5 mm, and also reflux during the Valsalva maneuver are criteria for diagnosis of pelvic varicose veins.^23^^,^ 24

**Figure 2:**
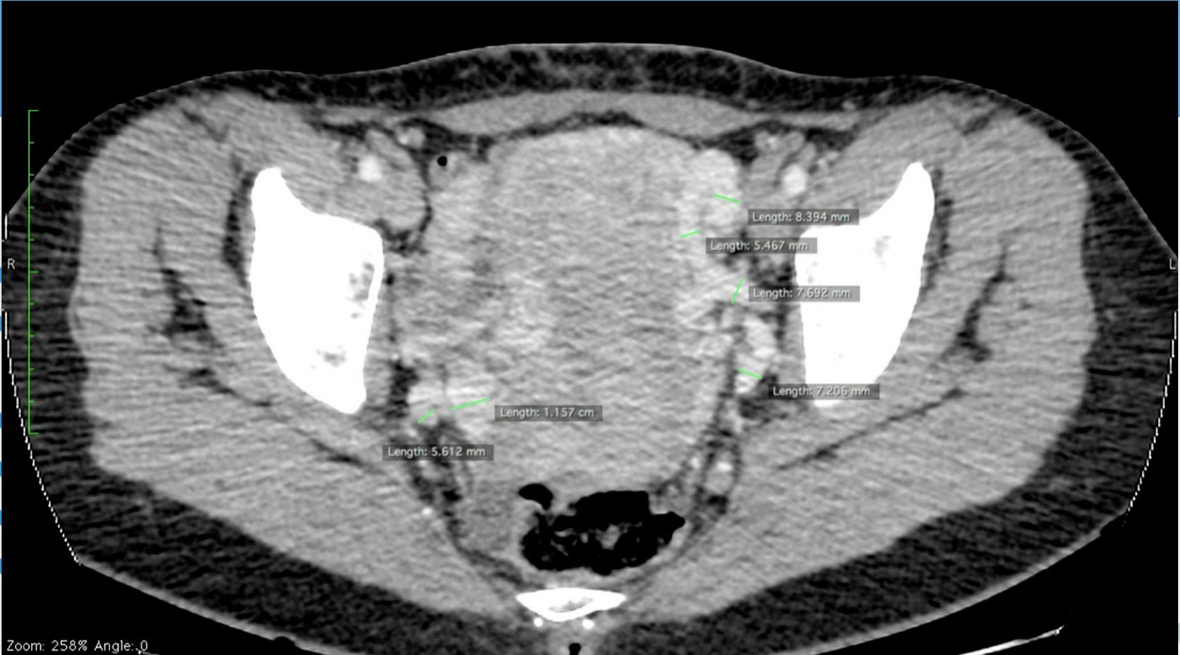
Axial angiotomography slice in venous phase showing several varicose parauterine veins of varying diameters, as large as 11 mm.

Magnetic resonance and angiotomography offer greater sensitivity for diagnosis of PCS, and also enable investigation of other abdominal venous compression syndromes.^25^^,^ 26 However, since they are performed with the patient in dorsal decubitus, the extent and diameter of the pelvic collateral network and dilation of the ovarian vein may be underestimated (Figure 2).^26^

**Figure 3:**
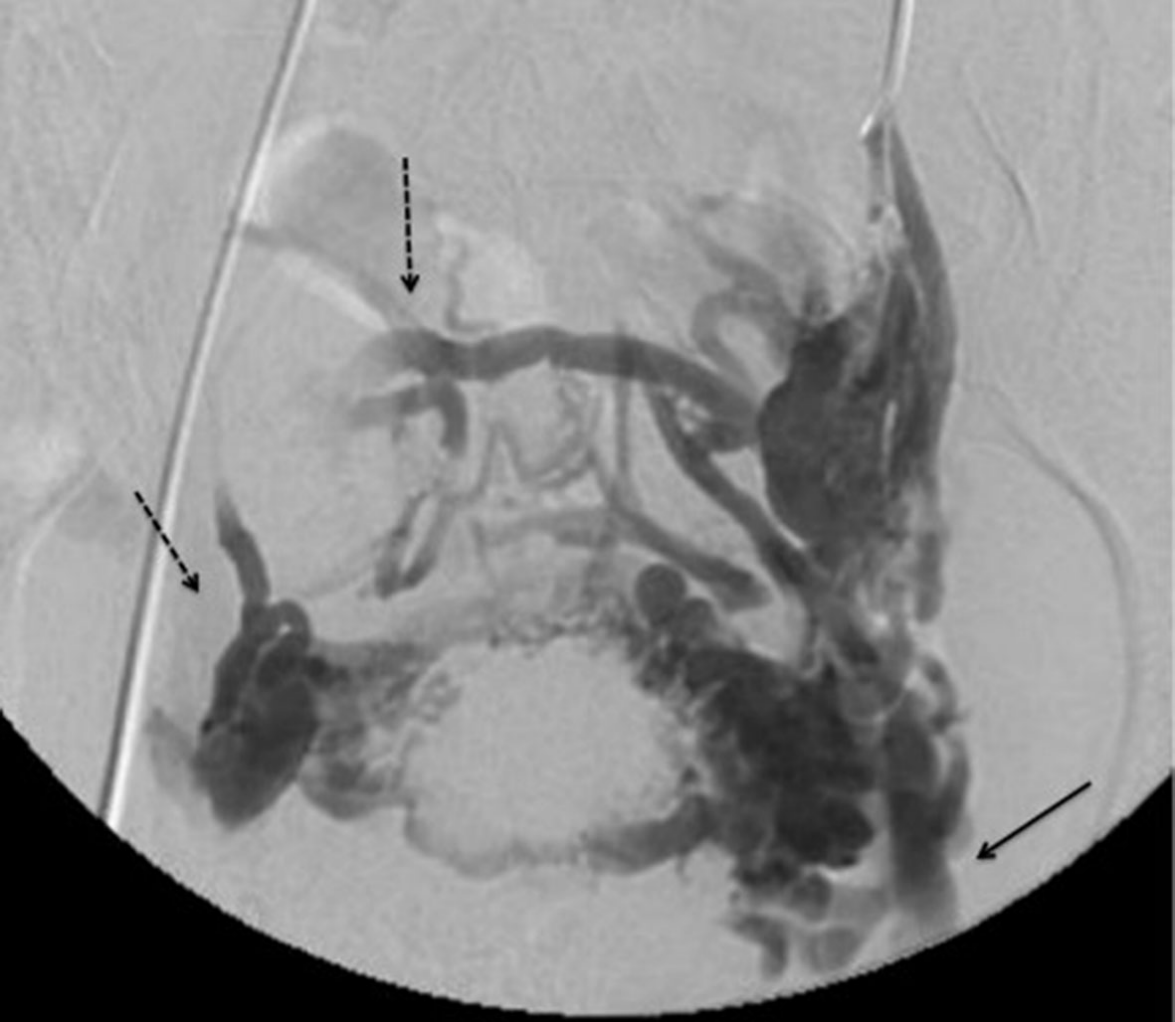
Pelvic phlebography during Valsalva maneuver, showing large varicose vessels. There is contrast reflux to the left common femoral vein (arrow) and the right parauterine complex (broken arrow).

Venography is the gold standard examination for diagnosis of PCS (Figure 3). It may show tortuous and dilated veins in the myometrium which communicate with bilateral pelvic varicose veins, with diameter > 10 mm, slow blood flow (< 3 cm/s), and retrograde venous flow in the left ovarian vein. Since it is an invasive examination, venography should preferably be reserved for patients who require intervention or when diagnostic doubts remain.^27^

## TREATMENTS

### Clinical treatment

The objective of drug-based treatment is to suppress ovarian function and induce vasoconstriction of the dilated veins. Medroxyprogesterone acetate, gonadotropin-releasing hormone (GnRH) analogs, and venotonic agents for 6 months provoke partial relief from symptoms. However, long-term pharmacological therapy is not recommended for treatment of PCS because of the adverse symptoms and limited efficacy.^28^

Gavrilov et al. investigated the impact of 20-30 mmHg compression on the symptoms of PCS. They observed significant clinical improvement in 81.3% of the group treated with compressive shorts, with no clinical improvement or improved venous drainage of the pelvic organs associated with wearing elastic stockings.^29^

### Surgical treatment

Surgery is an option for cases that are refractory to other treatment methods and with symptoms that compromise daily activities. The most often used technique is laparoscopic transperitoneal ligature of the ovarian vein.^4^ Limiting factors include greater surgical mortality and an increased number of complications, such as deep venous thrombosis, retroperitoneal hematoma, and ileus.^3^

### Endovascular treatment

In 1993, Edwards et al. described the first case of bilateral embolization of the ovarian vein to treat PCS.^30^ Since then, countless case reports and cohort studies have been published, with a mean success rate of 75%.^31^

**Figure 4:**
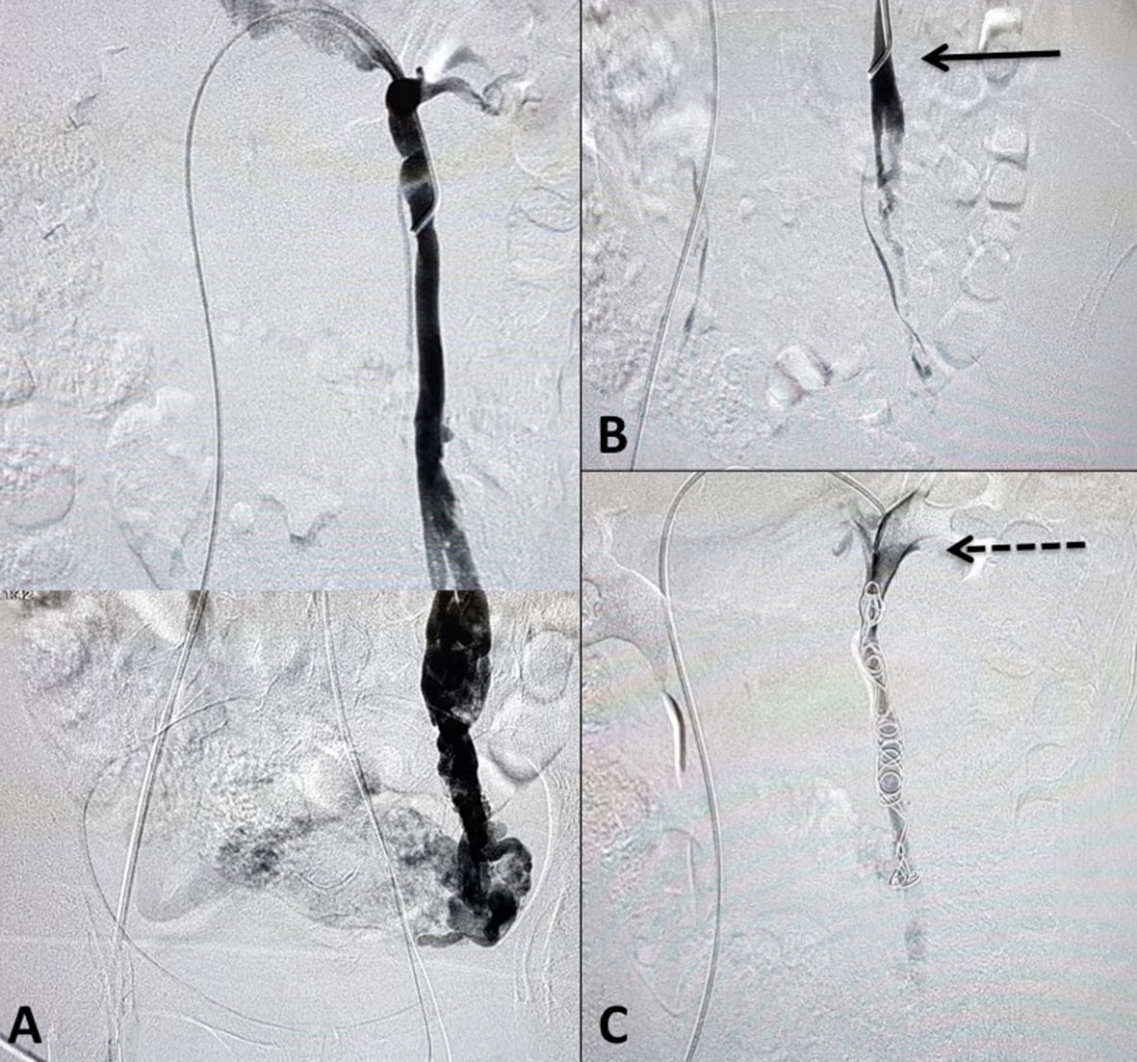
Reconstruction of left gonadal phlebography showing increased diameter and reflux to parauterine veins (A). After injection of polidocanol foam, the pelvic veins can no longer be seen (B), and the MPA2 catheter tip is maintained at the distal portion of the iliac bone, with the aim of preventing retrograde flow of foam to the gonadal vein (arrow). After embolization of the left gonadal vein with six 0.035” coils, the left ovarian vein is completely excluded (broken arrow (C)).

Access for embolization can be obtained via the right femoral vein or via the jugular, basilic, or cephalic veins. If access is obtained via the inferior vena cava, then Cobra 2 or Simmons 1 catheters are used to reach the renal vein. If the access employed is the superior vena cava, MPA2 catheters are recommended. After access, a long sheath can be guided to the renal vein to provide support. After left renal phlebography to identify reflux in the gonadal vein, this vein is catheterized. Phlebography of the gonadal vein should initially be performed at rest, to assess reflux along its entire length, and then during the Valsalva maneuver, to assess contralateral venous reflux and reflux to the lower limbs. Embolization is facilitated by use of the microcatheter and microcoils,^3^ but a 0.035” controlled-release coil system or plugs can also be used, depending on the experience of the surgeon and the availability of materials. The present authors prefer the 0.035” system. The average number of coils employed is six, but can vary from two to ten.^32^ Embolization is initiated in the pelvic veins, with the catheter positioned beyond the junction with the renogonadal collaterals, generally at the level of the lower half of the sacroiliac joint, maintaining the catheter in position to avoid reflux of the embolization agent to the gonadal vein. Next, the coils or plugs are released into the gonadal veins.^33^ Sclerotherapy of the hypogastric veins can also be performed. In men with varicocele, polidocanol and sodium tetradecyl sulfate can be employed as sclerosing agents (Figure 4).^4^ After embolization, the patient may suffer mild to moderate discomfort, which typically responds to non-steroidal anti-inflammatories.^34^

Veins with caliber greater than 12 mm increase the risk of coils migrating to the pulmonary artery, which is one of the main complications of the procedure.^35^ Other complications include venous perforation, local phlebitis, deep venous thrombosis, and reactions to the contrast, which occur in 3.4-4.4% of cases.^2^ Six weeks after embolization, echography should be performed again, to assess the degree of reflux remaining.^34^

There is still no evidence whether unilateral or bilateral embolization produces better outcomes.^33^ The treatment decision should therefore depend on the severity of symptoms, on the anatomy of the pelvic varicose veins, and on the degree of reflux.^36^

Many studies report greater than 80% reductions in pelvic varicose veins and symptoms after embolization.^37^ In a review covering 520 patients with a mean follow-up time of 15 months, 46% reported significant relief and 40.6% reported moderate relief from symptoms after embolization. The review compared the number of patients for whom treatment resulted in relief with the number who did not benefit, finding that 86.6% improved and 13.4% reported little or no relief.^17^ Asciutto et al.^4^ reported that conservative treatment of ovarian veins was associated with unfavorable prognosis, whereas patients with ovarian incompetence only exhibited clinical improvement after embolization (mean grade of 5.1 before and 2.1 after the procedure). Embolization can result in improvement of PCS in 91% of the patients and of lower limb varicose veins in 51%.^9^

In cases in which the response to embolization is not total, potentially related issues include variability in the details of the procedure, in patient characteristics, and the possibility that an analog visual pain scale fails to capture all of the benefits of the procedure.^2^

van der Vleuten et al.^38^ reported that 42% of patients required a second embolization and two patients underwent two additional procedures, with no effect on symptoms.

A systematic review conducted by Daniels evaluated efficacy in 1,308 patients in 22 cohorts, with no randomized clinical trials, finding a 75% mean rate of improvement of symptoms in the first 3 months. However, there was also improvement lasting for up to 45 months after the procedure.^31^

There are few studies comparing embolization with other treatments. Chung et al. demonstrated that embolization was superior to hysterectomy and oophorectomy for relief of PCS. The mean visual pain scale score reduced from 7.8 to 3.2 in the embolization group, contrasting with 4.6 in a bilateral oophorectomy group and 5.6 among patients who underwent unilateral oophorectomy.^39^

## NUTCRACKER SYNDROME

Clinical presentation of NCS includes lumbar pain and hematuria, caused by distension of Gerota’s fascia and blood leakage secondary to dilatation of the venules of the pyelocaliceal system.^8^ As the pathology progresses, venous hypertension causes dilatation of the left gonadal vein and valve incompetence, transmitting hypertension to the pelvic veins, which become dilated over time.^8^

In NCS, the pressure gradient between the left renal vein and the vena cava can exceed 3 mmHg.^17^ The peak velocities in the narrowed and distended portions of the left renal vein have 70-90% sensitivity, but vary depending on the position of the patient.^8^ A ratio between the dimensions of the narrowed and dilated portions of the left renal vein > 4.9 is used as a diagnostic criterion.^40^

Open surgery and endovascular treatment are both treatment options. Open surgery causes greater morbidity and longer duration renal ischemia.^8^ Complications after stent placement are related to migration to the right atrium or the ostium of the left renal vein and protrusion into the inferior vena cava. Larger stents are therefore recommended to avoid this complication.^41^ When PCS is the dominant clinical presentation, endovascular treatment should be employed, with or without embolization.^42^ However, there is no definition in the literature of the best method for treatment of NCS when it is associated with PCS. The decision on the best treatment should be based on local anatomy and also patient age.^3^

## CONCLUSIONS

Pelvic congestion syndrome is still an under-diagnosed pathology and one that causes the people affected considerable morbidity. Vascular surgeons can improve diagnosis of PCS by raising awareness among primary care professionals about the signs and symptoms of the pathology. Ultimately, PCS tends to be diagnosed by exclusion and so greater awareness about it will increase referrals of appropriate patients to specialists.

Endovascular treatment is the best option available for this pathology. Notwithstanding, the data in the literature are not based on evidence from randomized clinical trials. It still remains to identify the population that will most benefit from embolization and to develop measures for assessing the outcome that are more suited to the complexity of PCS.

## References

[1] Taylor HC (1949). Vascular congestion and hyperemia; their effect on function and structure in the female reproductive organs; the clinical aspects of the congestion-fibrosis syndrome. Am J Obstet Gynecol.

[2] Meissner MH, Gibson K (2015). Clinical outcome after treatment of pelvic congestion syndrome: sense and nonsense. Phlebology.

[3] O’Brien MT, Gillespie DL (2015). Diagnosis and treatment of the pelvic congestion syndrome. J Vasc Surg Venous Lymphat Disord.

[4] Asciutto G, Asciutto KC, Mumme A, Geier B (2009). Pelvic venous incompetence: reflux patterns and treatment results. Eur J Vasc Endovasc Surg.

[5] Lechter A, Lopez G, Martinez C, Camacho J (1991). Anatomy of the gonadal veins: a reappraisal. Surgery.

[6] Ahlberg NE, Bartley O, Chidekel N (1966). Right and left gonadal veins. An anatomical and statistical study. Acta Radiol Diagn (Stockh).

[7] Heinz A, Brenner E (2010). Valves of the gonadal veins: an anatomical study. Phlebologie.

[8] Kurklinsky AK, Rooke TW (2010). Nutcracker phenomenon and nutcracker syndrome. Mayo Clin Proc.

[9] Hartung O (2015). Embolization is essential in the treatment of leg varicosities due to pelvic venous insufficiency. Phlebology.

[10] Borghi C, Dell’Atti L (2016). Pelvic congestion syndrome: the current state of the literature. Arch Gynecol Obstet.

[11] Rastogi N, Kabutey NK, Kim D (2012). Incapacitating pelvic congestion syndrome in a patient with a history of May-Thurner syndrome and left ovarian vein embolization. Ann Vasc Surg.

[12] Daugherty SF, Gillespie DL (2015). Venous angioplasty and stenting improve pelvic congestion syndrome caused by venous outflow obstruction. J Vasc Surg Venous Lymphat Disord.

[13] Santoshi RKN, Lakhanpal S, Satwah V, Lakhanpal G, Malone M, Pappas PJ (2018). Iliac vein stenosis is an underdiagnosed cause of pelvic venous insufficiency. J Vasc Surg Venous Lymphat Disord.

[14] Phillips D, Deipolyi AR, Hesketh RL, Midia M, Oklu R (2014). Pelvic congestion syndrome: etiology of pain, diagnosis, and clinical management. J Vasc Interv Radiol.

[15] Moore CJ (2011). Pelvic Congestion Syndrome Update - Diagnostic and therapeutic options for this often unrecognized condition and one center’s approach to treatment. Endovascular Today.

[16] Ignacio EA, Dua IVR, Sarin S (2008). Pelvic congestion syndrome: diagnosis and treatment. Semin Intervent Radiol.

[17] Mahmoud O, Vikatmaa P, Aho P (2016). Efficacy of endovascular treatment for pelvic congestion syndrome. J Vasc Surg Venous Lymphat Disord.

[18] Herrera-Betancourt AL, Villegas-Echeverri JD, López-Jaramillo JD, López-Isanoa JD, Estrada-Alvarez JM (2018). Sensitivity and specificity of clinical findings for the diagnosis of pelvic congestion syndrome in women with chronic pelvic pain. Phlebology.

[19] Galego GN, Silveira PG, Bortoluzzi CT, Franklin RN, Ronchi TM (2015). Síndrome da Congestão Venosa Pélvica e resultados do tratamento endovascular: série de casos. J Vasc Bras.

[20] Gültaşli NZ, Kurt A, Ipek A (2006). The relation between pelvic varicose veins, chronic pelvic pain and lower extremity venous insufficiency in women. Diagn Interv Radiol.

[21] Labropoulos N, Jasinski PT, Adrahtas D, Gasparis AP, Meissner MH (2017). A standardized ultrasound approach to pelvic congestion syndrome. Phlebology.

[22] Nascimento AB, Mitchell DG, Holland G (2002). Ovarian veins: magnetic resonance imaging findings in an asymptomatic population. J Magn Reson Imaging.

[23] Malgor RD, Spentzouris G, Adrahtas D, Gasparis AP, Tassiopoulos AK, Labropoulos N (2013). The role of duplex ultrasound in the pelvic congestion syndrome workup. J Vasc Surg Venous Lymphat Disord.

[24] Malgor RD, Adrahtas D, Spentzouris G, Gasparis AP, Tassiopoulos AK, Labropoulos N (2014). The role of duplex ultrasound in the workup of pelvic congestion syndrome. J Vasc Surg Venous Lymphat Disord.

[25] Yang DM, Kim HC, Nam DH, Jahng GH, Huh CY, Lim JW (2012). Time-resolved MR angiography for detecting and grading ovarian venous reflux: comparison with conventional venography. Br J Radiol.

[26] Arnoldussen CW, de Wolf MA, Wittens CH (2015). Diagnostic imaging of pelvic congestive syndrome. Phlebology.

[27] Leiber LM, Thouveny F, Bouvier A (2014). MRI and venographic aspects of pelvic venous insufficiency. Diagn Interv Imaging.

[28] Knuttinen MG, Xie K, Jani A, Palumbo A, Carrillo T, Mar W (2015). Pelvic venous insufficiency: imaging diagnosis, treatment approaches, and therapeutic issues. AJR Am J Roentgenol.

[29] Gavrilov SG, Karalkin AV, Turischeva OO (2018). Compression treatment of pelvic congestion syndrome. Phlebology.

[30] Edwards RD, Robertson IR, MacLean AB, Hemingway AP (1993). Case report: pelvic pain syndrome--successful treatment of a case by ovarian vein embolization. Clin Radiol.

[31] Daniels JP, Champaneria R, Shah L, Gupta JK, Birch J, Moss JG (2016). Effectiveness of embolization or sclerotherapy of pelvic veins for reducing chronic pelvic pain: a systematic review. J Vasc Interv Radiol.

[32] Hocquelet A, Le Bras Y, Balian E (2014). Evaluation of the efficacy of endovascular treatment of pelvic congestion syndrome. Diagn Interv Imaging.

[33] Maleux G, Stockx L, Wilms G, Marchal G (2000). Ovarian vein embolization for the treatment of pelvic congestion syndrome: long-term technical and clinical results. J Vasc Interv Radiol.

[34] Lopez AJ (2015). Female pelvic vein embolization: indications, techniques, and outcomes. Cardiovasc Intervent Radiol.

[35] Yamasaki W, Kakizawa H, Ishikawa M (2012). Migration to the pulmonary artery of nine metallic coils placed in the internal iliac vein for treatment of giant rectal varices. Acta Radiol Short Rep.

[36] Kim HS, Malhotra AD, Rowe PC, Lee JM, Venbrux AC (2006). Embolotherapy for pelvic congestion syndrome: long-term results. J Vasc Interv Radiol.

[37] Bittles MA, Hoffer EK (2008). Gonadal vein embolization: treatment of varicocele and pelvic congestion syndrome. Semin Intervent Radiol.

[38] van der Vleuten CJ, van Kempen JA, Schultze-Kool LJ (2012). Embolization to treat pelvic congestion syndrome and vulval varicose veins. Int J Gynaecol Obstet.

[39] Chung MH, Huh CY (2003). Comparison of treatments for pelvic congestion syndrome. Tohoku J Exp Med.

[40] Kim KW, Cho JY, Kim SH (2011). Diagnostic value of computed tomographic findings of nutcracker syndrome: correlation with renal venography and renocaval pressure gradients. Eur J Radiol.

[41] Hartung O, Grisoli D, Boufi M (2005). Endovascular stenting in the treatment of pelvic vein congestion caused by nutcracker syndrome: lessons learned from the first five cases. J Vasc Surg.

[42] Perkov D, Vrkić Kirhmajer M, Novosel L, Popić Ramač J (2016). Transcatheter ovarian vein embolisation without renal vein stenting for pelvic venous congestion and nutcracker anatomy. Vasa.

