# Supplementary material for: Pelvic congestion syndrome and embolization of pelvic varicose veins
Source: J Vasc Bras. 2019 Nov 8;18:e20190061. doi: 10.1590/1677-5449.190061 (PMC6852702; doi:10.1590/1677-5449.190061)
Supplement: Translation

# Translation: Síndrome da congestão pélvica e embolização de varizes pélvicas

**Authors:** Mateus Picada Corrêa, Larissa Bianchini, Jaber Nashat Saleh, Rafael Stevan Noel, Julio Cesar Bajerski

**Affiliations:** 1 Instituto Vascular de Passo Fundo – INVASC, Passo Fundo, RS, Brasil.

**Keywords:** varizes, insuficiência venosa, embolização

## Abstract

A síndrome da congestão pélvica (SCP) é definida como dor pélvica crônica há mais de 6 meses associada a varizes perineais ou vulvares, resultantes do refluxo ou obstrução das veias gonadais, glúteas ou periuterinas. A SCP é responsável por 16-31% dos casos de dor pélvica crônica, sendo diagnosticada sobretudo na terceira e quarta décadas de vida. Nos últimos anos, houve um interesse maior nessa patologia por parte dos cirurgiões vasculares devido à sua associação com insuficiência venosa de membros inferiores. Apesar de prevalente, a SCP ainda é pouco diagnosticada tanto nos consultórios ginecológicos quanto nos de angiologistas. Portanto, neste artigo revisaremos a etiologia e o diagnóstico desta patologia e os resultados dos diversos tipos de tratamentos disponíveis.

## INTRODUÇÃO

A existência de varizes pélvicas foi primeiramente descrita por Richet, em 1857, e o termo síndrome de congestão venosa da pelve foi cunhado por Taylor^1^ em 1949.

A síndrome da congestão pélvica (SCP) é definida como dor pélvica crônica há mais de 6 meses associada a varizes perineais ou vulvares, resultantes do refluxo ou da obstrução das veias gonadais, glúteas ou periuterinas. A SCP é responsável por 16-31% dos casos de dor pélvica crônica^2^, sendo diagnosticada sobretudo na terceira e quarta décadas de vida^3^. Nos últimos anos, houve um interesse maior nessa patologia por parte dos cirurgiões vasculares, devido à sua associação com insuficiência venosa de membros inferiores.

Apesar de prevalente, a SCP ainda é pouco diagnosticada tanto nos consultórios ginecológicos quanto nos de angiologistas e cirurgiões vasculares. Neste artigo, revisaremos a etiologia e o diagnóstico dessa patologia e os resultados dos diversos tipos de tratamentos disponíveis^4^.

## ANATOMIA

O sistema venoso do útero e do ovário é drenado para as veias ilíacas internas e gonadais. As veias pudendas recebem as tributárias parietais e tributárias viscerais do plexo gonadal e vesicovaginal e deságuam nas veias ilíacas internas. As veias ovarianas drenam o paramétrio, o cérvice, a mesossalpinge e o plexo pampiniforme, formando um rico plexo venoso-anastomótico^5^. A veia ovariana esquerda é formada pela união de duas ou três tributárias que se unem ao nível da quarta vértebra lombar, desaguando na veia renal esquerda à esquerda e na veia cava inferior à direita (Figura 1)^3^. Em 10% dos casos, a veia ovariana direita drena na veia renal direita^3^. O diâmetro médio das veias ovarianas é menor do que 5 mm^6^, e em 15% dos casos a veia gonadal esquerda não possui válvulas^3^. Porém, caso estas existam, podem ser encontradas sobretudo na porção distal da veia. Heinz e Brenner^7^, em estudo com 31 cadáveres, encontraram um caso de varizes pélvicas com válvulas presentes, e em nenhum dos 15 indivíduos com ausência de válvulas havia dilatação venosa. Entretanto, a incompetência valvular das veias gonadais pode estar presente em até 40% dos casos^8^.

**Figura 1:** Representação esquemática da anatomia das veias pélvicas. Ao = aorta; VCI = veia cava inferior; VRE = veia renal esquerda; VRD = veia renal direita; VOE = veia ovariana esquerda; VOD = veia ovariana direita; POD = plexo ovariano direito; POE = plexo ovariano esquerdo; PUD = plexo uterino direito; PUE = plexo uterino esquerdo.

## FISIOPATOLOGIA

Existem duas classificações etiológicas das varizes pélvicas. Uma delas classifica essas varizes em três tipos: tipo 1, por patologia da parede da veia, como insuficiência valvar, agenesia de válvula, malformações; tipo 2, secundária a compressões vasculares como síndrome do quebra-nozes (SQN), síndrome de May-Thurner (SMT) e por colateralização secundária à doença pós-trombótica; e tipo 3, secundária a compressão extrínseca local causada por patologias como endometriose ou massa tumoral^9^. A segunda classificação descreve quatro distúrbios: varizes vulvares sem SCP, insuficiência isolada da veia hipogástrica, refluxo primário das veias gonadais e colateralização pélvica secundária a síndromes compressivas ou compressões extrínsecas^3^.

Na SCP de causa primária, as varizes resultam do refluxo causado por válvulas incompetentes ou pela degeneração da parede venosa. O refluxo pode também ser resultante da compressão da veia renal esquerda pela artéria mesentérica superior, da compressão da veia ilíaca interna esquerda na SMT, ou de uma má posição uterina^10^, ou ser resultante de mudanças no padrão de fluxo decorrentes de hipertensão venosa à montante^8^. No homem, as varizes podem causar varicocele^11^. Daugherty e Gillespie^12^ encontraram compressão moderada a severa da veia ilíaca comum esquerda em 18 pacientes e um alto grau de estenose da veia cava inferior suprarrenal em um paciente. Em todos os pacientes, os sintomas pélvicos eram a queixa mais proeminente, e houve melhora destes após o implante de stent. Ademais, há relatos de algum grau de estenose da veia ilíaca em até 80% dos pacientes com insuficiência venosa pélvica^13^.

A etiologia do refluxo primário ainda não foi totalmente elucidada. Estima-se que até 50% dos pacientes apresentem algum componente genético^14^. O fator hormonal também parece ser um contribuinte, na medida em que o estradiol induz dilatação seletiva das veias ovarianas e uterinas durante a gestação, ocasionando maior estresse sobre as válvulas^15^. De fato, até 50% das mulheres com SCP apresentam ovários policísticos à ecografia, sem hirsutismo ou amenorreia^16^. A dor pélvica provém da estase sanguínea nas veias pélvicas dilatadas, que pode ativar receptores de dor das paredes dos vasos, além de promover a liberação de neurotransmissores e da substância P^14^.

## APRESENTAÇÃO CLÍNICA

Classicamente, o sintoma mais prevalente é a dor pélvica, que pode ser associada a dismenorreia, dispareunia e irritação vesical. Ao exame físico, há presença de varicosidades vulvares, das veias suprapúbicas e na face posterior das coxas^3^. Mahmoud et al.^17^ encontraram, em revisão de 20 estudos, a dismenorreia como sintoma referido em 86% (18,4-100%) das pacientes; além de dispareunia (40,8%), varicosidades em membros inferiores (58,7%) e varicosidades vulvares (45,9%). Sensibilidade à palpação do ovário e dispareunia apresentam sensibilidade de 94% e especificidade de 77% para SCP^18^.

Após serem descartadas outras causas mais comuns de dor pélvica crônica, incluindo endometriose, doença inflamatória pélvica, cistite intersticial e leiomiomas, realiza-se ecografia pélvica para visualização dos vasos gonadais^19^.

A associação de SCP com insuficiência venosa dos membros inferiores foi demonstrada por um estudo realizado na Turquia, o qual demonstrou que a SCP era a causa de dor pélvica crônica em 30% de 100 pacientes consecutivas, e que, destas, 70% apresentavam sintomas de insuficiência venosa de membros inferiores, com refluxo de femoral comum sendo o achado mais frequente^20^.

## EXAMES DE IMAGEM

O primeiro exame a ser realizado costuma ser a ecografia pélvica transvaginal^21^. A medida de dilatação de veia pélvica associada a dor pélvica é variável, sendo 4 mm considerada normal, 4-8 mm associada com refluxo assintomático, e > 8 mm associada com refluxo e sintomas^22^. Assim, são critérios para o diagnóstico de varizes pélvicas a visualização de veias ovarianas dilatadas, com diâmetro superior a 8 mm, e de veias parauterinas > 5 mm, e também a presença de refluxo durante a manobra de Valsalva^23^^,^ 24.

A ressonância magnética e a angiotomografia apresentam maior sensibilidade para o diagnóstico da SCP, além de permitirem a avaliação concomitante da presença de outras síndromes venosas compressivas abdominais^25^^,^ 26. Todavia, como são realizados com o paciente em decúbito dorsal, a extensão e o diâmetro da rede de colaterais pélvicas e a dilação da veia ovariana podem ser subestimados (Figura 2)^26^.

**Figura 2:** Corte axial de uma angiotomografia em fase venosa demonstrando diversas varizes parauterinas de diversos diâmetros, alcançando até 11 mm.

A venografia é o exame padrão-ouro para o diagnóstico da SCP (Figura 3). Ela pode evidenciar veias tortuosas e dilatadas no miométrio que se comunicam com varizes pélvicas bilaterais, com diâmetro > 10 mm, fluxo sanguíneo lentificado (< 3 cm/s,) e fluxo venoso retrógrado na veia ovariana esquerda. Por ser um exame invasivo, a venografia deve ser realizada preferencialmente nos pacientes que necessitem de intervenção ou na dúvida diagnóstica^27^.

**Figura 3:** Flebografia pélvica em Valsalva demonstrando grandes vasos varicosos. Há refluxo de contraste para a veia femoral comum esquerda (seta) e para o plexo parauterino direito (seta pontilhada).

## TRATAMENTOS

### Tratamento clínico

O objetivo da terapia medicamentosa é suprimir a função ovariana e induzir vasoconstrição das veias dilatadas. Acetato de medroxiprogesterona, análogos do hormônio liberador da gonadotrofina (GnRH) e venotônicos por 6 meses promovem alívio parcial dos sintomas. Porém, devido aos sintomas adversos e à eficácia limitada, a terapia farmacológica não é recomendada a longo prazo para o tratamento da SCP^28^.

Gavrilov et al. investigaram o impacto da compressão 20-30 mmHg nos sintomas da SCP. Foi encontrada melhora clínica significativa em 81,3% do grupo tratado com *shorts* compressivos, sem melhora clínica e nem da drenagem venosa dos órgãos pélvicos com o uso de meias elásticas^29^.

### Tratamento cirúrgico

A cirurgia é uma alternativa para os pacientes refratários a outras modalidades de tratamento e que apresentam sintomas que prejudiquem as atividades diárias. A técnica mais utilizada é a ligadura laparoscópica transperitoneal da veia ovariana^4^. Entre seus fatores limitantes, destacam-se uma maior mortalidade cirúrgica e maior número de complicações, como trombose venosa profunda, hematoma retroperitoneal e íleo paralítico^3^.

### Tratamento endovascular

Em 1993, Edwards et al.^30^ descreveram o primeiro caso de embolização bilateral de veia ovariana para o tratamento de SCP. Desde então, inúmeros relatos de casos e coortes já foram publicados, com taxas médias de sucesso de 75%^31^.

O acesso para a embolização pode ser realizado tanto pela veia femoral direita como pelas veias jugular, basílica ou cefálica. Caso o acesso obtido seja pela veia cava inferior, cateteres Cobra 2 ou Simmons 1 são utilizados para acesso à veia renal. Se o acesso utilizado tiver sido a veia cava superior, cateteres MPA2 são recomendados. Após obtenção do acesso, uma bainha longa pode ser guiada até a veia renal para suporte. Após flebografia renal esquerda para identificação de refluxo na veia gonadal, esta é cateterizada. A flebografia de veia gonadal deve ser realizada inicialmente em repouso, para avaliação de refluxo em toda a sua extensão, e sob manobra de Valsalva, para avaliação do refluxo venoso contralateral e para os membros inferiores. A embolização é facilitada pelo sistema de microcateteres e micromolas^3^; porém, pode ser utilizado sistema de molas de destacamento controlado 0.035” ou plugs, conforme a experiência do cirurgião e disponibilidade dos materiais. Os autores preferem utilizar sistemas 0.035”. O número médio de molas utilizadas por procedimento é seis, podendo variar de duas a dez^32^. Inicia-se a embolização das veias pélvicas com o cateter posicionado após a junção das colaterais renogonadas, geralmente a nível da metade inferior da articulação sacroilíaca, mantendo o cateter em posição para evitar refluxo do agente embolizante para a veia gonadal. A seguir, as molas ou plugs são liberados nas veias gonadais^33^. A escleroterapia das veias hipogástricas também pode ser realizada. Em homens com varicocele, o polidocanol e o sódio tetradecil sulfato são agente esclerosantes que podem ser utilizados (Figura 4)^4^. Após a embolização, o paciente pode apresentar um desconforto leve a moderado por até 5 dias, que tipicamente responde a anti-inflamatórios não esteroides^34^.

**Figura 4:** Reconstrução de flebografia de gonadal esquerda demonstrando aumento de diâmetro e refluxo para veias parauterinas (A). Após injeção de espuma de polidocanol, as veias pélvicas não são mais visualizadas (B), e a ponta do cateter MPA2 mantém-se na parte distal do osso ilíaco com o intuito de evitar o fluxo retrógrado da espuma até a veia gonadal (seta). Após a embolização da veia gonadal esquerda com seis molas 0.035”, há completa exclusão da veia ovariana esquerda (seta pontilhada (C)).

Veias de calibre superior a 12 mm aumentam o risco da migração das molas para a artéria pulmonar, uma das principais complicações do procedimento^35^. Outras complicações incluem perfuração venosa, flebite local, trombose venosa profunda e reações ao contraste, que ocorrem em 3,4-4,4% dos casos^2^. Após 6 semanas da embolização, deve ser realizada uma nova ecografia, para avaliar o grau de refluxo remanescente^34^.

Ainda não há evidências que a embolização uni ou bilateral promova melhor desfecho^33^. Logo, a decisão sobre o tratamento deve depender da severidade dos sintomas, da anatomia das varizes pélvicas e do grau de refluxo^36^.

Diversos estudos reportam uma redução maior que 80% nas varizes pélvicas e nos sintomas após a embolização^37^. Em revisão com 520 pacientes com acompanhamento médio de 15 meses, 46% relataram alívio significativo e 40,6% alívio moderado dos sintomas após a embolização. Nos estudos dessa revisão, que compararam o número de pacientes que apresentaram alívio com aqueles que não se beneficiaram, a melhora foi de 86,6%, em contraponto a 13,4% que referiram pouco ou nenhum alívio^17^. Asciutto et al.^4^ referiram que o tratamento conservador das veias ovarianas é associado com um prognóstico desfavorável, enquanto que em pacientes com incompetência ovariana isolada há melhora clínica após a embolização (escore médio de 5,1 antes e 2,1 após o procedimento). A embolização pode gerar melhora da SCP em 91% dos pacientes e das varizes de membros inferiores em 51%^9^.

Nos casos em que há uma resposta incompleta à embolização, questões potencialmente relacionadas são a variabilidade nos detalhes do procedimento, nas características do paciente e também a falha das escalas analógicas visuais de dor em capturar todos os benefícios do procedimento^2^.

van der Vleuten et al.^38^ relataram que 42% dos pacientes necessitaram de uma segunda embolização e dois deles foram submetidos a mais dois procedimentos, sem efeito sobre seus sintomas.

Na revisão sistemática conduzida por Daniels, foi avaliada a eficácia em 1.308 pacientes de 22 coortes, sem ensaios clínicos randomizados, com melhora média de 75% dos sintomas nos primeiros 3 meses. Porém, ainda há uma melhora que se estende por até 45 meses após o procedimento^31^.

Existem poucos estudos comparando a embolização com outras terapias. Chung e Huh^39^ demonstraram que a embolização foi superior a histerectomia e ooforectomia para alívio da SCP. O escore médio visual de dor diminuiu de 7,8 para 3,2 no grupo da embolização, em contraponto a 4,6 no grupo da ooforectomia bilateral e a 5,6 nos pacientes submetidos a ooforectomia unilateral.

## SÍNDROME DO QUEBRA-NOZES

O quadro clínico da SQN inclui dor lombar e hematúria, resultantes da distensão da fáscia de Gerota e do extravasamento sanguíneo secundário à dilatação das vênulas do sistema pielocalicial^8^. Com a evolução da patologia, a hipertensão venosa causa dilatação da veia gonadal esquerda e insuficiência valvular, projetando a hipertensão para as veias pélvicas, que se tornam dilatadas com o passar do tempo^8^.

O gradiente pressórico entre a veia renal esquerda e a veia cava na SQN atinge mais de 3 mmHg^17^. Os picos de velocidade das porções estreitadas e distendidas da veia renal esquerda apresentam sensibilidade de 70-90%, porém variam conforme a posição do paciente^8^. A razão entre as dimensões da veia renal esquerda estreitada e sua porção dilatada é utilizada como critério diagnóstico quando for > 4,9^40^.

A cirurgia aberta e o tratamento endovascular constituem opções terapêuticas. A cirurgia aberta gera maior morbidade e maior tempo de isquemia renal^8^. Complicações após a implantação de stent relacionam-se à sua migração para o átrio direito ou para o óstio da veia renal esquerda e à sua protrusão na veia cava inferior. Assim, stents maiores são recomendados para evitar essa complicação^41^. Quando a SCP é a apresentação clínica dominante, deve ser realizado o tratamento endovascular, com ou sem embolização^42^. Não há, todavia, definição da literatura da melhor forma de tratamento da SQN quando associada à SCP. A decisão sobre a melhor terapia deve ser baseada na anatomia local e também na idade do paciente^3^.

## CONCLUSÃO

A SCP é uma patologia ainda subdiagnosticada e que gera grande morbidade para os indivíduos acometidos. Os cirurgiões vasculares podem melhorar o diagnóstico da SCP por meio da conscientização dos profissionais da atenção primária sobre os sinais e sintomas dessa patologia. Afinal, a SCP costuma ser um diagnóstico de exclusão, e maior conhecimento sobre ela aumentaria o referenciamento dos pacientes apropriados ao especialista.

A terapêutica endovascular é o tratamento disponível mais adequado para essa patologia. Apesar disso, os dados presentes na literatura não são baseados em evidências provenientes de ensaios clínicos randomizados. Ainda é necessário identificar a população que mais se beneficia da embolização e desenvolver medidas para avaliar o desfecho que melhor traduzam a complexidade da SCP.
